# Stochastic Model for Energy Propagation in Disordered Granular Chains

**DOI:** 10.3390/ma14071815

**Published:** 2021-04-06

**Authors:** Kianoosh Taghizadeh, Rohit Kumar Shrivastava, Stefan Luding

**Affiliations:** 1 Multi-Scale Mechanics, Faculty of Engineering Technology, MESA+, University of Twente, 7522NB Enschede, The Netherlands; s.luding@utwente.nl; 2 Institute of Applied Mechanics (CE), SC SimTech, University of Stuttgart, 70569 Stuttgart, Germany; 3 Shell Technology Center Bangalore, Seismic Analytics, Bengaluru 562149, Karnataka, India; rohit.kumar.shrivastava@gmail.com

**Keywords:** wave propagation, 1D granular chain, stochastic model, master equation, disorder

## Abstract

Energy transfer is one of the essentials of mechanical wave propagation (along with momentum transport). Here, it is studied in disordered one-dimensional model systems mimicking force-chains in real systems. The pre-stressed random masses (other types of disorder lead to qualitatively similar behavior) interact through (linearized) Hertzian repulsive forces, which allows solving the deterministic problem analytically. The main goal, a simpler, faster stochastic model for energy propagation, is presented in the second part, after the basic equations are re-visited and the phenomenology of pulse propagation in disordered granular chains is reviewed. First, the propagation of energy in space is studied. With increasing disorder (quantified by the standard deviation of the random mass distribution), the attenuation of pulsed signals increases, transiting from ballistic propagation (in ordered systems) towards diffusive-like characteristics, due to energy localization at the source. Second, the evolution of energy in time by transfer across wavenumbers is examined, using the standing wave initial conditions of all wavenumbers. Again, the decay of energy (both the rate and amount) increases with disorder, as well as with the wavenumber. The dispersive ballistic transport in ordered systems transits to low-pass filtering, due to disorder, where localization of energy occurs at the lowest masses in the chain. Instead of dealing with the too many degrees of freedom or only with the lowest of all the many eigenmodes of the system, we propose a stochastic master equation approach with reduced complexity, where all frequencies/energies are grouped into bands. The mean field stochastic model, the matrix of energy-transfer probabilities between bands, is calibrated from the deterministic analytical solutions by ensemble averaging various band-to-band transfer situations for short times, as well as considering the basis energy levels (decaying with the wavenumber increasing) that are not transferred. Finally, the propagation of energy in the wavenumber space at transient times validates the stochastic model, suggesting applications in wave analysis for non-destructive testing, underground resource exploration, etc.

## 1. Introduction

Disorder or heterogeneityexists at all spatial scales in nature, whether it is soil or matter in space. Disorder in granular materials (like soil) can manifest in many ways from the grain level to the system level (contact disorder, geometrical disorder, asphericity, layering, etc.). All may have an effect on the mechanical wave transmission through the granular material in its own unique way (for instance, contact disorder due to tiny polydispersity can reduce the mechanical wave speed and the transport of high-frequency waves [1,2,3,4,5,6,7]. Knowing these effects can aid us in numerous ways for subsurface exploration or for non-destructive testing of materials [8,9,10,11].

The diffusive (scattering) characteristics of momentum and energy transport during mechanical wave propagation are the focus of many ongoing investigations [12,13,14,15,16]. Predicting the energy propagation characteristics in the real and wavenumber space through disordered (simplified) model granular media, like chains, can assist in understanding the overall properties of wave propagation through real inhomogeneous media like soil; this eventually can assist in seismic prospecting, non-destructive testing, or designing metamaterials [17].

Energy attenuation of mechanical wave packets occurs due to various mechanisms, which can be broadly classified into two categories: scattering attenuation and intrinsic attenuation [18,19,20]. Scattering attenuation is the decrease in energy due to the spreading of energy across different frequencies, i.e., the decay of energy due to the re-direction of vibration. Intrinsic attenuation is the loss of energy by dissipative mechanisms such as mechanical heat generation. Isolating these mechanisms and understanding their effects independently have been the goal of wave propagation models. One-dimensional modeling assists in removing geometric/directional effects, while analyzing energy transfer across frequencies [21]. Some of the models to estimate scattering attenuation were mentioned in [22].

Including disorder, e.g., adding inclusions with different properties or size, will lead to enhanced absorption in typical frequency ranges/bands, relative to the basis material. Thus, there is a need to study the effects of disorder individually, and hence, the focus of this article will only be on mass disorder, for which a 1D granular chain was chosen so that the P-wave mode is isolated from the shear or rotational mode [23,24]. A mechanical wave propagating through this simplified model 1D granular medium is bound to suffer from multiple scattering [25,26,27,28]. However, regardless of scattering, linear waves preserve some coherence that manifests as intensity correlations [29]. The results obtained from the chain also represent attributes of both longitudinal P-waves (compressional) and S-waves (shear) in a 3D system, as stated in [4,30]; the frequency filtering effects are very similar to those in a 3D system, as observed in [1]. This is all the more reason to study the energy content and spectral energy response of the propagating wave [17,31,32,33,34].

Classical continuum theories and effective medium theory experience difficulty in modeling wave propagation in the intermediate- or high-frequency range because of their inability to resolve the microstructure of the material [35,36]. Continuum numerical techniques like the Finite Element Method (FEM), Finite Difference Method (FDM), etc., can be used to predict wave propagation characteristics only if the right parameters are used, which are often difficult to find. However, the Discrete Element Method (DEM) [37] is a numerical technique that takes into account the disordered microstructure of the material and the nonlinear contact forces between the interacting constituent granules of the media. This microscopic description is detailed, but also costly, so that only small volumes can be modeled. Nevertheless, the DEM can be used to obtain the parameters of stochastic mesoscale models [38]. These then eventually can be used for continuum, macroscopic wave propagation analyses, hence paving the way towards a statistical micro-informed macroscopic treatment of the problem [39,40,41,42].

The dynamic wave propagation in a granular chain can be argued to be a Markovian process; the initial waveform (displacement/velocity of the particles) and the granular chain properties (pre-compression, sizes/masses of the particles through which the mechanical wave propagates) are sufficient to construct/predict successively the waveform at later time intervals [43,44,45]. The transition probability functions of the Markovian processes can be written in the form of the Chapman–Kolmogorov equation, one of the versions of this equation is the master Equation [46]. Hence, a master equation can be used to represent the transfer of energy across wavenumbers during mechanical wave propagation.

Complementing earlier studies on the transfer of energy between frequency bands [4], evolving in space [25,47,48,49], this research focuses also on the transfer of energy across different wavenumbers, as the system evolves in time. A master equation is devised and utilized for analyzing the transfer energy across different wavenumbers, studied with the aid of a one-dimensional granular chain [4,42,50,51]. Using the ensembled spatio-spectral energy response from the granular chain, the transfer coefficients of a reduced-complexity, disorder-specific master equation are evaluated. The proposed approach (master equation) features groups of frequencies in bands, instead of dealing with too many eigenmodes. This is different in spirit from reduced order modeling [52] since one accounts for all frequencies, also the largest ones, but gives up the details by grouping all modes with a similar frequency, gaining tremendous speedup without requiring running expensive numerical simulations, which eventually can be used for mean-field macroscopic/continuum analyses.

This paper is organized in the following manner. Section 2 gives the micro-mechanical model of the granular chain with the linearized Hertzian repulsive interaction force acting between the granules; two types of initial conditions (impulse propagation condition and standing wave condition; Section 2.5 and Section 2.6) are used to analyze energy propagation with distance and across wavenumbers. Section 2.7 gives a brief description about the mass disorder used in the granular chain. Section 3 lists the equations used for computing the total energy responses both in the real and wavenumber space. Section 3.4 formulates the master equation and proposes the procedures to evaluate the transfer rates of energy across the wavenumber space (components of the transfer matrix). Results are discussed in Section 4, and final conclusions are presented in Section 5.

## 2. Granular Chain Model

In this section, the equations of motion are derived by employing a general nonlinear force–displacement relation. A granular chain of mesoscopic granules/particles was modeled using the Hertzian repulsive interaction potential (a good approximation for spherical particles) [53,54]. The repulsive interaction force between adjacent particles *i* and *j* (*j* can be i+1 or i−1), with mass ${\tilde{m}}^{\left(i\right)}$ and ${\tilde{m}}^{\left(j\right)}$, is:

(1) $${\tilde{F}}_{\left(i,j\right)}={\tilde{\kappa }}_{\left(i,j\right)}{\tilde{\delta }}_{\left(i,j\right)}^{3/2},\;{\tilde{\delta }}_{\left(i,j\right)}\geq 0,$$

where ${\tilde{\kappa }}_{\left(i,j\right)}$ is the dimensional inter-particle contact stiffness, ${\tilde{\delta }}_{\left(i,j\right)}$ is the dimensional dynamic inter-particle overlap, and the 3/2 exponent is due to the Hertzian potential. The dimensional dynamic overlap is written as ${\tilde{\delta }}_{\left(i,j\right)}={\tilde{r}}^{\left(i\right)}+{\tilde{r}}^{\left(j\right)}-\left|{\tilde{x}}^{\left(i\right)}-{\tilde{x}}^{\left(j\right)}\right|$ such that it is strictly non-negative for contacts, where $\tilde{r}$ and $\tilde{x}$ are the absolute dimensional radius and position, respectively. Anticipating an appropriate scaling of the problem, the tilde symbol is used for dimensional quantities. The granular chain has a pre-confining force $\tilde{P}$ such that there is some initial strain associated with the equilibrium configuration, which prevents opening and closing of contacts. This assists in modeling mechanical wave propagation across well-established granular chains. Assuming an external pre-compressional force $\tilde{P}$ on the granular chain in mechanical equilibrium, the initial particle overlap is given by:

(2) $${\tilde{\Delta }}_{o}={\left(\frac{\tilde{P}}{{\tilde{\kappa }}_{\left(i,j\right)}{\tilde{\ell }}^{3/2}}\right)}^{2/3},$$

where $\tilde{\ell }$ is a length scale, i.e., characteristic length. To obtain a non-dimensionalized equation of motion for particles, the physical parameters have to be scaled. The minimum number of scaling parameters required for arriving at a non-dimensionalized equation of motion are the characteristic mass (${\tilde{m}}_{o}$), which we take as the mean particle mass of the system, the characteristic stiffness (${\tilde{\kappa }}_{o}$), and a length scale ($\tilde{\ell }$). Different choices can be selected for the length scale $\tilde{\ell }$, e.g., the particle size or the driving amplitude. Here, the length scale is related related to the overlap of a characteristic contact in a static equilibrium configuration. For the characteristic stiffness, the contact of two identical particles with the mean mass is chosen. The initial overlap between these particles under the pre-compressive loading condition becomes $\tilde{\ell }={\tilde{\Delta }}_{o}$ (with non-dimensional initial overlap $\Delta_{o}={\tilde{\Delta }}_{o}/\tilde{\ell }=1$). Inserting the scaled particle overlap $\delta_{\left(i,j\right)}={\tilde{\delta }}_{\left(i,j\right)}/\tilde{\ell }$ in Equation (1) yields:

(3) $${\tilde{F}}_{\left(i,j\right)}={\tilde{\kappa }}_{\left(i,j\right)}{\tilde{\ell }}^{3/2}\delta_{\left(i,j\right)}^{3/2},$$

The non-dimensional mass $b^{\left(i\right)}={\tilde{m}}^{\left(i\right)}/{\tilde{m}}_{o}$; the non-dimensional stiffness is $\kappa_{\left(i,j\right)}={\tilde{\kappa }}_{\left(i,j\right)}/{\tilde{\kappa }}_{o}$; and the non-dimensional displacement is $u=\tilde{u}/\tilde{\ell }$. Without new scaling parameters, this also defines the non-dimensional time $t=\tilde{t}/{\tilde{t}}_{c}$ where:

(4) $${\tilde{t}}_{c}=\sqrt{\frac{{\tilde{m}}_{o}}{{\tilde{\kappa }}_{o}{\tilde{\ell }}^{1/2}}};$$

thus, the non-dimensional repulsive interaction force becomes:

(5) $$F_{\left(i,j\right)}=\frac{{\tilde{t}}_{c}^{2}}{{\tilde{m}}_{o}\tilde{\ell }}{\tilde{F}}_{\left(i,j\right)}.$$

An equation of motion for the particle *i* (*i* = 1,..., N) is written as:

(6) $${\tilde{m}}^{\left(i\right)}\frac{d^{2}{\tilde{x}}^{\left(i\right)}}{d{\tilde{t}}^{2}}={\tilde{\kappa }}_{\left(i-1,i\right)}{\tilde{\ell }}^{3/2}\delta_{\left(i-1,i\right)}^{3/2}-{\tilde{\kappa }}_{\left(i,i+1\right)}{\tilde{\ell }}^{3/2}\delta_{\left(i,i+1\right)}^{3/2}.$$

The displacement of particle *i* from its equilibrium configuration ${\tilde{x}}_{0}^{\left(i\right)}$ becomes ${\tilde{u}}^{\left(i\right)}=\tilde{\ell }u^{\left(i\right)}={\tilde{x}}^{\left(i\right)}-{\tilde{x}}_{0}^{\left(i\right)}$. Hence, the scaled overlap between *i* and *j* (with j>i) is $\delta_{\left(i,j\right)}=\Delta +u^{\left(i\right)}-u^{\left(j\right)}$.

The non-dimensional equation of motion for particle *i* is now given by:

(7) $$\begin{array}{cc} b^{\left(i\right)}\frac{d^{2}u^{\left(i\right)}}{dt^{2}} & =F_{\left(i-1,i\right)}-F_{\left(i,i+1\right)}\\ \; & =\kappa_{\left(i-1,i\right)}{\left[\Delta_{\left(i-1,i\right)}-\left(u^{\left(i\right)}-u^{\left(i-1\right)}\right)\right]}^{3/2}-\kappa_{\left(i,i+1\right)}{\left[\Delta_{\left(i,i+1\right)}-\left(u^{\left(i+1\right)}-u^{\left(i\right)}\right)\right]}^{3/2}, \end{array}$$

where the stiffness ratio $\kappa_{\left(i,j\right)}=\kappa_{\left(\tilde{i},j\right)}/{\tilde{\kappa }}_{0}$ was defined implicitly. Equation (7) can be solved numerically using the Verlet integration scheme, and it can be used for analyses related to nonlinear dynamics of particles (Hertzian).

### 2.1. Linearized Equation of Motion

Here, we linearize the general force–displacement relation about the equilibrium configuration. The nondimensional phrasing of Equation (1) is given by:

(8) $$F_{\left(i,j\right)}=\kappa_{\left(i,j\right)}\delta_{(i,j)\prime }^{3/2}$$

which can be expanded around the initial overlap $\Delta_{\left(i,j\right)}$ as:

(9) $$F_{\left(i,j\right)}=\kappa_{\left(i,j\right)}\Delta_{\left(i,j\right)}^{3/2}+\frac{3}{2}\kappa_{\left(i,j\right)}\Delta_{\left(i,j\right)}^{1/2}\left(\delta_{\left(i,j\right)}-\Delta_{\left(i,j\right)}\right)+\frac{3}{8}\kappa_{\left(i,j\right)}\Delta_{\left(i,j\right)}^{-1/2}{\left(\delta_{\left(i,j\right)}-\Delta_{\left(i,j\right)}\right)}^{2}+...$$

If the amplitudes of displacement $u^{\left(i\right)}$ are small during mechanical wave propagation, so the relative displacements $\delta_{\left(i,j\right)}-\Delta_{\left(i,j\right)}=u^{\left(i\right)}-u^{\left(j\right)}$, and the nonlinear terms can be ignored so that:

(10) $$F_{\left(i,j\right)}≅\kappa_{\left(i,j\right)}\Delta_{\left(i,j\right)}^{3/2}-\frac{3}{2}\kappa_{\left(i,j\right)}\Delta_{\left(i,j\right)}^{1/2}\left(u^{\left(j\right)}-u^{\left(i\right)}\right).$$

Hence, the linearized equation of motion for particle *i* becomes:

(11) $$\begin{array}{cc} b^{\left(i\right)}\frac{d^{2}u^{\left(i\right)}}{dt^{2}} & =\kappa_{\left(i-1,i\right)}\Delta_{\left(i-1,i\right)}^{1/2}\left[\Delta_{\left(i-1,i\right)}-\frac{3}{2}\left(u^{\left(i\right)}-u^{\left(i-1\right)}\right)\right]\\ \; & -\kappa_{\left(i,i+1\right)}\Delta_{\left(i,i+1\right)}^{1/2}\left[\Delta_{\left(i,i+1\right)}-\frac{3}{2}\left(u^{\left(i+1\right)}-u^{\left(i\right)}\right)\right], \end{array}$$

which can eventually be written as:

(12) $$b^{\left(i\right)}\frac{d^{2}u^{\left(i\right)}}{dt^{2}}=\frac{3}{2}\kappa_{\left(i,i+1\right)}^{2/3}\left(u^{\left(i+1\right)}-u^{\left(i\right)}\right)-\frac{3}{2}\kappa_{\left(i-1,i\right)}^{2/3}\left(u^{\left(i\right)}-u^{\left(i-1\right)}\right).$$

There are i=0,...,N+1 (in total N+2) particles in the granular chain with 0^*th*^ and ${\left(N+1\right)}^{th}$ particles as the boundaries of the chain such that $u^{\left(0\right)}=0$ and $u^{\left(N+1\right)}=0$. Alternatively, periodic boundaries are realized by using N+1 particles, equivalent to setting $u^{\left(0\right)}=u^{\left(N+1\right)}\neq 0$, which is also allowed to move, so all vectors and matrices get length N+1 with $2^{i}=N+1$, with the *i* integer. Equation (12) results in *N* equations, which are assembled in a matrix form:

(13) $$M\frac{d^{2}u}{dt^{2}}=Ku,$$

where M is a diagonal matrix with $b^{\left(1\right)},b^{\left(2\right)},b^{\left(3\right)}....b^{\left(N\right)}$ as diagonal elements, K is a symmetric, tri-diagonal matrix with $-\frac{3}{2}\left(\kappa_{\left(i+1,i\right)}^{2/3}+\kappa_{\left(i-1,i\right)}^{2/3}\right)$ as diagonal, $\frac{3}{2}\kappa_{\left(i+1,i\right)}^{2/3}$ as superdiagonal, and $\frac{3}{2}\kappa_{\left(i-1,i\right)}^{2/3}$ as subdiagonal elements, and other elements of K are zero. u is the displacement vector containing displacements $u^{\left(i\right)}$ as elements. In this study, the focus is on the effect of mass disorder only. Therefore, all coupling stiffnesses are independent of the contact ($\kappa_{\left(i,j\right)}=1$). Thus, initial overlaps become the same $\Delta_{\left(i,j\right)}=1$. Considering this assumption, the stiffness matrix diagonal components become −2, and the sub- and super-diagonal components are +1.

### 2.2. Neglect of Contact Damping

First, we consider the conservation of energy to facilitate new model development. Intrinsic attenuation at a contact point is expressed by damping, which is proportional to the relative velocities of the particles. Contact damping at the microscopic scale happens due to the viscous dissipation of energy, which is velocity dependent when particles are deformed. Furthermore, if liquid bridges are formed at the contact points, this happens. Damping of grain motions is included as standard in the DEM calculations. Although damping is a physical reality and a physically meaningful mechanism, our concern here is not to include a source of dissipation, i.e., conservation of energy for new model development. This allows us to not only have a continuous oscillation, but also, it simplifies the solution of the equations of motion.

Considering a damping force in contact would change the differential equation, Equation (13), to:

(14) $$M\frac{d^{2}u}{dt^{2}}+D\frac{du}{dt}-Ku=0,$$

leading to the difficulties of solving the second-order differential equation system where D is the damping matrix. This problem is called the quadratic eigenvalue problem for the (complex) eigenfrequencies. The complexity of solving the second-order differential equation for granular packings was explained in [55,56].

Furthermore, it must be noted that since the applied displacement is much smaller than the initial overlap, particles will not obtain large relative velocities, which reveals that the damping force will be negligible for small amplitude waves in the elastic, jammed regime. Hence, it makes sense to avoid the damping term completely and solve a differential equation, which gives non-complex answers. However, if an applied displacement amplitude is greater than the initial overlap, $u^{\left(i\right)}-u^{\left(j\right)}\geq \Delta$ ($\delta_{\left(i,j\right)}\leq 0$), then openings of contacts will occur, which means contacts between particles can open or break. In all cases presented, we applied small enough displacements to make sure that particles never break the chain.

### 2.3. Implication of Mass-Disorder in a Monodisperse Chain

Earlier, the effect of contact stiffness disorder and nonlinearity on the transmission of signals in one-dimensional pre-stressed systems subjected to a harmonic perturbation of the boundary was studied [3].

By creating a monodisperse (size) chain of particles with mass disorder, the effects of this contact disorder (as present in the Hertzian model) are removed. This way, only the effect of mass disorder is considered. We assigned $\kappa_{\left(i,j\right)}=1$ and $\Delta_{\left(i,j\right)}=1$ for all contacts (i,j) in the equations of motion given by Equation (7), which is a straightforward task in numerical simulation. From the experimental perspective, such a monodisperse, mass-disordered chain is created by removing material from the particles centers or the inclusion of denser cores. This mass change should not raise a problem for the contact stiffness. Eventually, there is no issue for the Hertz model, which is based on deformations at the contacting surfaces.

### 2.4. Linear Model Solution

To solve the equation of motion under the imposed boundary conditions, Equation (13) is stated in its eigenvector basis. This gives *N* independent relations. After the transformation into the eigensystem, the eigenvectors and eigenfrequencies associated with Equation (13) are determined. Assuming $A=-M^{-1}K$ (not symmetric, in general), Equation (13), arrives at the known eigenvalue problem:

(15) $$\frac{d^{2}u}{dt^{2}}=-\mathrm{Au}.$$

Using an ansatz $u=u^{\prime }\exp^{I\omega t}$, Equation (15) becomes an eigenvalue problem:

(16) $$Au=\omega^{2}u.$$

The eigenvalues $\omega_{j}^{2}$ ($\omega_{j}$ are the natural frequencies) and the eigenvectors $s_{\left(j\right)}$ of the matrix A represent the eigendomain of the dynamic granular chain. The set of eigenvectors ($s_{\left(j\right)}$) can be orthonormalized by the condition:

(17) $$s_{\left(i\right)}^{T}Ms_{\left(j\right)}=\delta_{ij},$$

where $\delta_{ij}$ is the Kronecker delta symbol. A matrix S is constructed using $s_{\left(j\right)}$ as columns and arranged in such a manner that their corresponding $\omega_{j}$ are in increasing order. S is an eigenbasis matrix and can be used for projecting u into the eigenspace by the relation:

(18) $$z=S^{-1}u,$$

where z is the vector of amplitudes (per eigenmode) in the eigenspace. Using the transformation $S^{-1}\mathrm{AS}=G$, where G is a diagonal matrix with $\omega_{j}^{2}$ as the diagonal elements, Equation (15) becomes:

(19) $$\frac{d^{2}z}{dt^{2}}=-\mathrm{Gz}.$$

**Figure 1:**
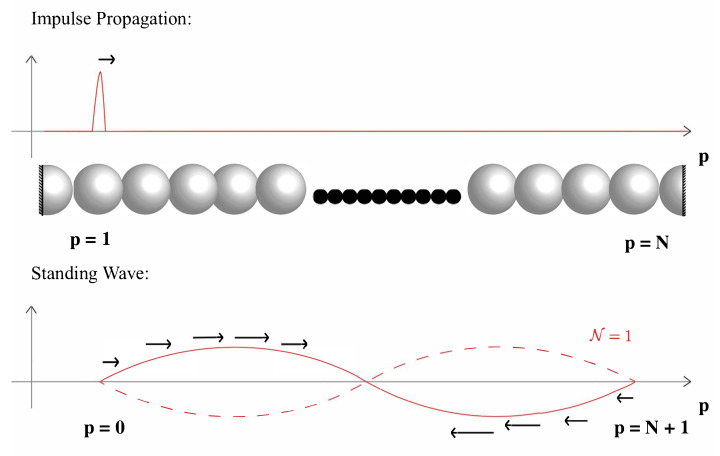
Impulse and (sinusoidal) standing wave initial conditions for a fixed-end and periodic granular chain, respectively.

The solution of this equation is given by (in the eigenspace and real space, respectively)

(20) $$z\left(t\right)=C^{\left(1\right)}a+C^{\left(2\right)}b\;\mathrm{or}\;u\left(t\right)=SC^{\left(1\right)}a+SC^{\left(2\right)}b,$$

where $C^{\left(1\right)}$ is a diagonal matrix with $\sin(\omega_{j}t)$ as diagonal elements, $C^{\left(2\right)}$ is also a diagonal matrix with $\cos(\omega_{j}t)$ as diagonal elements, a and b are vectors, which are determined from initial conditions $u_{o}$ (initial displacement vector) and $v_{o}$ (initial velocity vector).

(21) $$a=H^{-1}S^{-1}v_{o}\;\mathrm{and}\;b=S^{-1}u_{o},$$

where H is a diagonal matrix with $\omega_{j}$ as the diagonal elements. Two different types of initial conditions are used for different types of analyses in the upcoming sections, impulse propagation and standing wave analysis, see Figure 1.

### 2.5. Impulse Propagation Condition

The initial condition for impulse propagation requires $u_{o}$ and $v_{o}$ to be:

(22) $$u^{\left(i\right)}\left(t=0\right)=0,\;v^{\left(i\neq n\right)}\left(t=0\right)=0,\;v^{\left(n\right)}\left(t=0\right)=v_{o},$$

where *n* is the particle number to which the impulse is imparted. The condition $v_{o}\ll 1$, initial particle overlap $\Delta_{o}$, avoids opening and closing of contacts to maintain the validity of the linearized equations of motion (Section 2.1). The first or the center particles in a granular chain were imparted with an impulse for further analyses in Section 4.1. Equation (22) is used to get:

(23) $$a=H^{-1}S^{-1}v_{o}\;\mathrm{and}\;b=0.$$

Hence, the displacement and velocity of particle *i* become:

(24) $$u=SC^{\left(1\right)}H^{-1}S^{-1}v_{o}\;\mathrm{and}\;v=SC^{\left(2\right)}S^{-1}v_{o};$$

which is also written as:

(25) $$u^{\left(i\right)}\left(t\right)=v_{o}\sum_{j=1}^{N}\frac{S_{ij}S_{nj}\sin\left(\omega_{j}t\right)}{\omega_{j}}\;\mathrm{and}\;v^{\left(i\right)}\left(t\right)=v_{o}\sum_{j=1}^{N}S_{ij}S_{nj}\cos\left(\omega_{j}t\right).$$

### 2.6. Standing Wave Condition

For studying standing waves in the (periodic) chain, an initial sinusoidal waveform is given to the chain in the form of $u_{o}=u_{o}\sin\left(N\frac{2\pi p}{N+1}\right)$ and $v_{o}=0$ (where p=1,...,N+1 or 0,...,N, N=1, …, (N+1)/2 specifies the particular tone of the standing wave). sin can be replaced easily by a cos, since N=(N+1)/2 does not work with sin. The condition $u_{o}\ll 1$ (initial particle overlap $\Delta_{o}$) avoids opening and closing of contacts to maintain the validity of the linearized equations of motion (Section 2.1). a and b are given as:

(26) $$a=H^{-1}S^{-1}v_{o}=0\;\mathrm{and}\;b=S^{-1}u_{o}.$$

Hence, the displacement and velocity of the particles become:

(27) $$u=SC^{\left(2\right)}S^{-1}u_{o}\;\mathrm{and}\;v=-\mathrm{SH}C^{\left(1\right)}S^{-1}u_{o};$$

which is also written as:

(28) $$\begin{array}{cc} u^{\left(i\right)}\left(t\right) & =u_{0}\sum_{j=1}^{N}S_{ij}\cos\left(\omega_{j}t\right)\sum_{p=1}^{N}S_{pj}\sin\left(N\frac{2\pi i}{N+1}\right),\\ v^{\left(i\right)}\left(t\right) & =-u_{0}\sum_{j=1}^{N}\omega_{j}S_{ij}\sin\left(\omega_{j}t\right)\sum_{p=1}^{N}S_{pj}\sin\left(N\frac{2\pi i}{N+1}\right). \end{array}$$

For completeness, we note that the solution for arbitrary initial conditions is the superposition of Equations (24) and (27).

### 2.7. Mass Disorder/Disorder Parameter (ξ) and Ensembles

The diagonal elements of the mass matrix M, $b^{\left(1\right)},b^{\left(2\right)},b^{\left(3\right)},...b^{\left(N\right)}$, were selected from a normal distribution $f^{\left(n\right)}\left(b\right)=\frac{1}{\xi \sqrt{2\pi }}e^{-\frac{{\left(b-1\right)}^{2}}{2\xi^{2}}}$; the standard deviation (ξ) quantifies the disorder parameter of the granular chain; and the scaled average of the distribution is normalized to unity [57]. A similar model was used previously in [4,50,57] for various wave propagation analyses. It was observed in [50] that the shape of the disorder probability (binary, normal, uniform, or any other distribution) produces quantitatively similar wave propagation effects (frequency filtering, attenuation, or mechanical wave velocities), up to a certain strength of disorder. Furthermore, disorder in mass or stiffness, or both, did not change the observations, so that we only studied mass disorder here. The physical quantities (e.g., displacement, velocity, total energy, etc.) of multiple realizations of granular chains with a particular disorder parameter were averaged to obtain ensembled quantities, depicted by angular brackets 〈...〉.

## 3. Energy Evolution and the Master Equation Model

For calculating the kinetic energy of individual elements/particles, we define the matrix $KE_{pq}$ where:

(29) $$\mathrm{KE}=\frac{1}{2}M\left[v⊗v\right]=\frac{1}{2}{\mathrm{Mvv}}^{T}.$$

The kinetic energy of individual elements/particles is the diagonal elements of the matrix KE, i.e., $KE_{PP}$ (capital letters PP are used as indices to denote the diagonal elements to avoid confusion with pp, which implies the summation of the diagonal elements, i.e., the trace of the matrix KE):

(30) $$KE^{\left(p\right)}\left(t\right)=KE_{PP}=\delta_{pP}\delta_{qP}KE_{pq}=\frac{1}{2}b^{\left(p\right)}{\left(v^{\left(p\right)}\left(t\right)\right)}^{2}.$$

The total kinetic energy is the trace of the matrix KE, i.e., $KE_{pp}$, (see Appendix A)

(31) $$KET\left(t\right)=KE_{pp}=\delta_{pq}KE_{pq}=\frac{1}{2}\sum_{p=1}^{N}b^{\left(p\right)}{\left(v^{\left(p\right)}\left(t\right)\right)}^{2}.$$

The potential energy of individual elements is basically the energy stored in the form of compression at the contacts of an element; it arises from the forces ($F^{\left(p\right)}$) exerted by other elements in contact with the element whose potential energy is being examined. For the potential energy, as well, we define the matrix:

(32) $$\mathrm{PE}=-\frac{1}{2}K\left[u⊗u\right]=-\frac{1}{2}{\mathrm{Kuu}}^{T}=-\frac{1}{2}{\mathrm{Fu}}^{T},\;\mathrm{with}\;F=\mathrm{Ku},$$

where K is the stiffness matrix (Equation (13)). The potential energy for individual elements is the diagonal elements of the matrix PE,

(33) $$PE^{\left(p\right)}\left(t\right)=PE_{PP}=\delta_{pP}\delta_{qP}PE_{pq}=-\frac{1}{2}F^{\left(p\right)}\left(t\right)u^{\left(p\right)}\left(t\right).$$

The total potential energy is the trace of the matrix PE, i.e., $PE_{pp}$, (see Appendix A)

(34) $$PET\left(t\right)=PE_{pp}=\delta_{pq}PE_{pq}=-\frac{1}{2}\sum_{p=1}^{N}F^{\left(p\right)}\left(t\right)u^{\left(p\right)}\left(t\right).$$

The total energy of individual particles is the sum of its kinetic and potential energies. Using Equations (29) and (32),

(35) $$\mathrm{TE}=\mathrm{KE}+\mathrm{PE}=KE_{pq}+PE_{pq}.$$

Using Equations (30) and (33),

(36) $$TE^{\left(p\right)}\left(t\right)=TE_{PP}=\delta_{pP}\delta_{qP}TE_{pq}=\frac{1}{2}\left(b\left(p\right)\right){\left(v^{\left(p\right)}\left(t\right)\right)}^{2}-\frac{1}{2}F^{\left(p\right)}\left(t\right)u^{\left(p\right)}\left(t\right).$$

We calculate the energies (potential energy, kinetic energy, and hence, total energy) relative to the initial pre-compressed state so that only the energy associated with wave propagation across the elements is taken into account.

The center of total energy is defined as [27]:

(37) $$R\left(t\right)=\frac{1}{TE_{tot}}\sum_{p=1}^{N}pTE^{\left(p\right)}\left(t\right)\;\mathrm{with}\;TE_{tot}=\sum_{p=1}^{N}TE^{\left(p\right)}\left(t\right).$$

The mean squared width of the propagating and trapped wave is [27]:

(38) $$r^{2}\left(t\right)=\frac{1}{TE_{tot}}\sum_{p=1}^{N}{\left(p-R\left(t\right)\right)}^{2}TE^{\left(p\right)}\left(t\right).$$

### 3.1. Energy Conservation

The energy of the system (chain) can be calculated by vector multiplications at a particular instance of time; the non-unitary dimension of the vector gives the respective information of the individual particles. Starting from the impulse initial condition in Section 2.5, using $v=SC^{\left(2\right)}Ha$ and the orthonormality condition $S^{T}MS=I$, where I is the identity matrix, the kinetic energy of the chain at a particular instant of time becomes:

(39) $$\begin{array}{cc} E_{kin}\left(t\right) & =\frac{1}{2}{\left(SC^{\left(2\right)}Ha\right)}^{T}M\left(SC^{\left(2\right)}Ha\right)\\ \; & =\frac{1}{2}a^{T}H^{T}{\left(C^{\left(2\right)}\right)}^{T}S^{T}MSC^{\left(2\right)}Ha=\frac{1}{2}a^{T}H{\left\{C^{\left(2\right)}\right\}}^{2}Ha=\frac{1}{2}\sum_{j}a_{j}^{2}\omega_{j}^{2}\sin^{2}\left(\omega_{j}t\right). \end{array}$$

Since $C^{\left(1\right)}$, $C^{\left(2\right)}$, and H are diagonal matrices, their transpositions are equal to the original. Note that there is no summation convention applied here. On the other hand, using $u=SC^{\left(1\right)}a$, $v=SC^{\left(2\right)}Ga$ and the orthonormality condition, the potential energy of the chain at a particular instant of time can be written as:

(40) $$\begin{array}{cc} E_{pot}\left(t\right) & =-\frac{1}{2}u^{T}Ku\\ \; & =-\frac{1}{2}u^{T}M\frac{d^{2}u}{dt^{2}}\\ \; & =-\frac{1}{2}{\left(SC^{\left(1\right)}a\right)}^{T}M\frac{dv}{dt}\\ \; & =-\frac{1}{2}{\left(SC^{\left(1\right)}a\right)}^{T}M\frac{dSC^{\left(2\right)}Ha}{dt}\\ \; & =\frac{1}{2}a^{T}C^{\left(1\right)}S^{T}MSC^{\left(1\right)}{\left\{H\right\}}^{2}a\\ \; & =\frac{1}{2}a^{T}H{\left\{C^{\left(1\right)}\right\}}^{2}Ha=\frac{1}{2}\sum_{j}a_{j}^{2}\omega_{j}^{2}\cos^{2}\left(\omega_{j}t\right). \end{array}$$

Hence, the total energy becomes a sum over all eigenmode energies:

(41) $$E_{tot}\left(t\right)=E_{kin}\left(t\right)+E_{pot}\left(t\right)=\frac{1}{2}\sum_{j}a_{j}^{2}\omega_{j}^{2}.$$

We can see that $E_{tot}$ is independent of time, which means the energy of the chain is conserved. If the summation term is dropped, Equation (41) gives the energy of the eigenmodes of the chain; thus, $E_{tot}\left(\omega_{j}\right)=\frac{1}{2}a_{j}^{2}\omega_{j}^{2}$.

### 3.2. Total Energy in the Wavenumber Domain

TE or $TE_{pq}$ is in the real space; to transform it into the wavenumber space $\hat{\mathrm{TE}}$ or ${\hat{TE}}_{km}$ (*k* and *m* being rows and columns in wavenumber space), there is a need to change the basis as $\hat{\mathrm{TE}}=\mathrm{DFT}\;\mathrm{TE}\;{\mathrm{DFT}}^{-1}$, where DFT is the discrete Fourier transform matrix (DFT can be computed numerically as the dftmt(x) matrix in MATLAB, where x is the size of the square matrix DFT. Note that DFT is an orthogonal matrix; hence, ${\mathrm{DFT}}^{-1}$ = ${\mathrm{DFT}}^{H}$, where superscript *H* denotes the conjugate transpose of a matrix.), which can be used to calculate the Fourier transform of vectors such that $\hat{u}=\mathrm{DFT}\;u$ and $\hat{v}=\mathrm{DFT}\;v$, where $\hat{u}$ and $\hat{v}$ are displacement and velocity vectors in the wavenumber space, respectively. Hence,

(42) $$\hat{\mathrm{TE}}=\mathrm{DFT}\;\mathrm{TE}\;{\mathrm{DFT}}^{-1},$$

with the trace yielding the total energy in the wavenumber space per eigenmode wavenumber *k*:

(43) $$TE^{\left(k\right)}\left(t\right)={\hat{TE}}_{KK}=\delta_{kK}\delta_{mK}{\hat{TE}}_{km}.$$

### 3.3. Binning Energy

Before establishing a numerical master equation, we first bin the total energy calculated in the wavenumber space. In order to not deal with so many eigenmodes, instead, one group is considered with an averaged energy for the group. The binning is done by:

(44) $$e^{\left(r\right)}\left(t\right)=\sum_{r-\Delta k/2}^{r+\Delta k/2}TE^{\left(k\right)}\left(t\right),$$

where Δk is the bandwidth of the bin and *r* is the central wavenumber.

The total energy is conserved; hence,

(45) $$E_{tot}\left(t\right)=\sum_{r=1}^{B}e^{\left(r\right)}\left(t\right)=\sum_{k}TE^{\left(k\right)}\left(t\right),$$

where *B* is the total number of bins assigned in the wavenumber space. The binned spectral energy is normalized by the total energy as a probability density:

(46) $${\hat{e}}^{\left(r\right)}\left(t\right)=\frac{1}{E_{tot}\left(t\right)}e^{\left(r\right)}\left(t\right),$$

so that $\sum_{r}{\hat{e}}^{\left(r\right)}\left(t\right)=1$.

A homogeneous distribution of energy thus corresponds to constant ${\hat{e}}^{\left(r\right)}={\hat{e}}_{B}=1/B$, i.e., the inverse number of bins.

The initial condition for inserting a single energy into a single wavenumber bin *k* is ${\hat{e}}^{\left(r\right)}\left(0\right)=\delta_{rk}$. For such systems, without disorder, ξ=0, the energy in the wavenumber space is invariant in time.

However, for increasing disorder, ξ>0, only a decreasing fraction of energy, ${\hat{e}}_{0}^{\left(r\right)}={\hat{e}}^{\left(r\right)}\left(0\right)\Psi \left(\xi ,r\right)$, remains in the original band *r*. Therefore, more and more “free” energy becomes available for being transferred to other wavenumbers, ${\hat{\hat{e}}}^{\left(r\right)}={\hat{e}}^{\left(r\right)}-{\hat{e}}_{0}^{\left(r\right)}$. The function:

(47) $$\Psi \left(\xi ,r\right)={\hat{e}}_{B}+\left(1-{\hat{e}}_{B}\right)\exp\left(-{\left[\xi r/2\right]}^{3/2}\right),$$

quantifies the fraction of energy in band *r*, which is not available for transfer, and is postulated from the solutions to the analytical chain model as $\Psi \left(\xi ,k\in r\right)=e^{\left(r\right)}\left(t\to \infty \right)/e^{\left(r\right)}\left(t=0\right)$, for energy inserted only into lower bands (note that for large bands, *r*, the long-time energy ${\hat{e}}^{\left(r\right)}\left(t\to \infty \right)$, cannot be directly used to estimate Ψ, since it contains also a continuous input from other bands, as discussed in the next subsection), with wavenumbers k≪π. For larger k≈π, the limit value $\Psi \left(\xi ,k\right)={\hat{e}}_{B}$ is postulated, based on the assumption that for large *k*, all bands are fed approximately the same amount of energy.

Finally, we note that the same Ψ also applies for energy inserted into multiple bands (not shown in this study).

### 3.4. Stochastic Master Equation

The master equation is an efficient tool in analyzing the stochastic crisscross transfer of energy between different wavenumber bands. In contrast to traditional, deterministic methods of order reduction [52,58,59,60,61,62], we did not focus on a subset of (lower) eigenmodes of the system, but grouped modes by wavenumbers (frequencies) to stochastically model the evolution in time, using only the much reduced set of wavenumber bands. Note that the master equation for frequency vs. space was already discussed in [4]. The transfer of spatio-spectral energy at time *t* is formulated as the evolution increment per time interval:

(48) $$\frac{d{\hat{e}}^{\left(s\right)}\left(t\right)}{dt}=\;Q_{ss}\;{\hat{\hat{e}}}^{\left(s\right)}\left(t\right)+\sum_{r\neq s}Q_{rs}\;{\hat{\hat{e}}}^{\left(r\right)}\left(t\right)\;,$$

with ${\hat{\hat{e}}}^{\left(s\right)}\left(t\right)={\hat{e}}^{\left(s\right)}\left(t\right)-{\hat{e}}_{0}^{\left(s\right)}$, where $Q_{ss}=-\sum_{r\neq s}Q_{sr}$ depicts the energy loss (rate) from a particular wavenumber band *s*, which eventually gets transferred to all other wavenumber bands r≠s. The non-diagonal $Q_{sr}$, or $Q_{rs}$, quantifies the transfer rates of energy from *s*, or *r*, to other wavenumber bands *r*, or *s*, respectively. The master equation in symbolic form relates the evolution of the energy vector, $\hat{e}$, to the transfer matrix product with the free, available energy $\hat{\hat{e}}$, such that:

(49) $$\frac{d\hat{e}}{dt}=\frac{d\hat{\hat{e}}}{dt}=Q\hat{\hat{e}}=Q\left(\hat{e}-{\hat{e}}_{0}\right)\;.$$

The short time evolution is linear in Q and $\hat{\hat{e}}$, whereas the long time evolution of the master equation results in the steady-state solution $Q\hat{\hat{e}}=0$.

### 3.5. Computing the Elements of Matrix Q

The elements of matrix Q can be computed numerically using the boundary condition in Section 2.6; a standing wave mode k∈s belonging to a particular wavenumber band *s* can be agitated in the form of a sinus (or cosinus) initial condition; see Equation (28).

The energy decay from normalized ${\hat{e}}_{0}^{\left(s\right)}=1$, or ${\hat{\hat{e}}}_{0}^{\left(s\right)}=1-\Psi \left(\xi ,s\right)$, can be fitted by $\langle {\hat{\hat{e}}}^{\left(s\right)}\left(t\right)\rangle /{\hat{\hat{e}}}_{0}^{\left(s\right)}=\left(1-y_{1}\right)e^{x_{1}\left(t-t_{0}\right)}+y_{1}$, where $x_{1}<0$ is the typical decay rate, at $t=t_{0}$, and $y_{1}$ represents the extrapolated saturation value (since $y_{1}$ is extrapolated forward in time, far beyond the narrow fit range, it is not the true terminal saturation). The brackets 〈...〉 indicate that ensembling in *k*-space is used to improve the quality of the fit. Similarly, $Q_{sr}$ is determined by fitting $\langle {\hat{\hat{e}}}^{\left(r\right)}\left(t\right)\rangle /{\hat{\hat{e}}}_{0}^{\left(s\right)}=y_{2}\left(1-e^{-x_{2}\left(t-t_{0}\right)}\right)$, where $x_{2}y_{2}$ represents the (positive) growth rate, at $t=t_{0}$, and $y_{2}$ represents the long-term saturation value.

Furthermore, one can apply Taylor expansion on the nonlinear fitting expressions to obtain a linear procedure: Considering the first term, avoiding higher orders, we can rewrite the expressions as: $\langle {\hat{\hat{e}}}^{\left(s\right)}\left(t\right)\rangle \approx 1+\left(1-y_{1}\right)x_{1}\left(t-t_{0}\right)$, with $Q_{ss}=\left(1-y_{1}\right)x_{1}$ the (negative) decay rate, at $t=t_{0}$, and $\langle {\hat{e}}^{\left(r\right)}\left(t\right)\rangle \approx x_{2}y_{2}\left(t-t_{0}\right)$, with $Q_{sr}=x_{2}y_{2}$. Hence, the energy decay of a particular wavenumber (band) as the energy spreads to other bands can be fit by $\langle {\hat{e}}^{\left(s\right)}\left(t\right)\rangle \approx {\hat{\hat{e}}}^{\left(s\right)}\left(t\right)\rangle /{\hat{\hat{e}}}_{0}^{\left(s\right)}\approx 1-\left(-Q_{ss}\right)t$ for small time intervals (well before reaching the saturation regime); similarly, $Q_{sr}$ is determined by fitting $\langle {\hat{e}}^{\left(r\right)}\left(t\right)\rangle \approx \langle {\hat{\hat{e}}}^{\left(r\right)}\left(t\right)\rangle /{\hat{\hat{e}}}_{0}^{\left(s\right)}\approx Q_{sr}t$. Later, the linear and nonlinear fit are called the Linear (L) and Nonlinear (NL) procedures, respectively (Note that the origin of time is not exactly at zero, due to swing-in on the time scale of $t_{c}$. Fit quality is improved by allowing the additional parameter $t\to t-t_{0}$, with $t_{0}\approx t_{c}$ and typically evaluating the nonlinear slopes at time $t\approx 2t_{0}$ or $3t_{0}$, not at t=0).

## 4. Results and Discussion

A *N* particle long granular chain has been used with impulse and standing wave initial conditions for analyses. Section 4.1 deals with energy propagation in a granular chain and the associated energy transfer in space. Section 4.2 deals with the analyses associated with energy transfer between different wavenumbers in time.

### 4.1. Energy Propagation with Distance

Two types of impulse initial conditions are used. In one of the systems, the first particle (N=1) was imparted with initial velocity $v_{o}$. In the other system, the center particle was imparted with $v_{o}$. Equations (37) and (38) were used for diffusion analyses associated with the impulse propagating in the granular chain.

#### 4.1.1. First Particle Excitation

**Figure 2:**
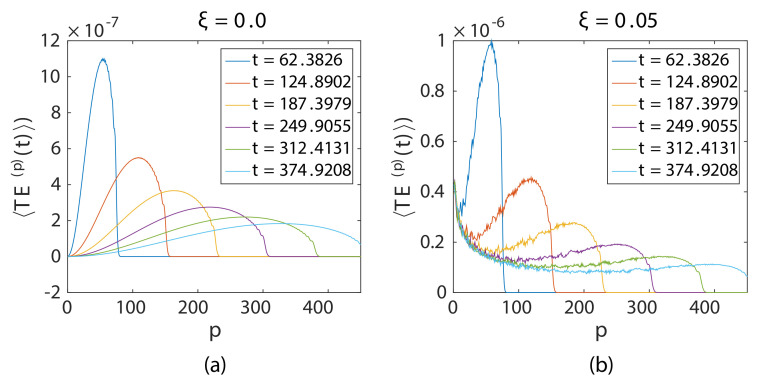
Ensembled total energy signal at different instances of time for (**a**) ordered and (**b**) disordered chains.

**Figure 3:**
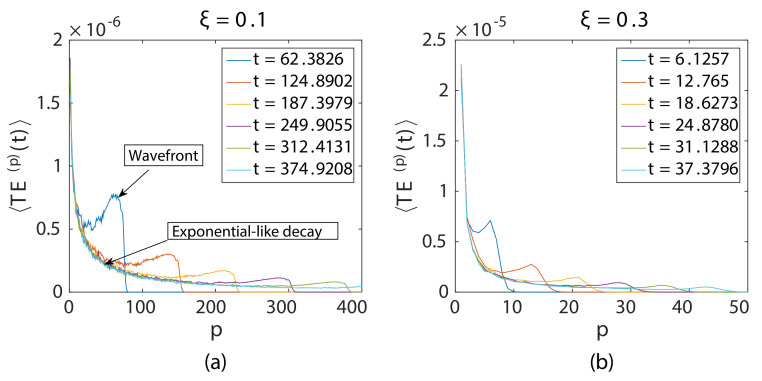
Ensembled total energy signal at different instances of time for moderate and strongly disordered chains.

Here, an N=1024 particle long granular chain was used. Particle p=1 was imparted with $v_{o}=0.01$. The time step for the computation was Δt=0.1250, and the maximum time evaluated was $t_{max}=1024$, chosen in order to avoid reflection of the incident wave from the boundary. Figure 2 and Figure 3 display the ensembled total energy signal of four different disordered chains ξ=0 (Figure 2a), ξ=0.05 (Figure 2b), ξ=0.1 (Figure 3a), and ξ=0.3 (Figure 3b). Five-hundred different realizations of chains were used for ensembling. It was observed that there were two peaks in the energy signal for all instances of time shown, irrespective of disorder except for the ordered chain (Figure 2a). The first peak was due to weak localization, a coherent backscattering effect during wave propagation near the source, and the second peak was due to the propagating coherent wavefront (Figure 3a). The ordered chain did not exhibit the weak localization peak because of the absence of disorder. Higher ξ showed a more rapid drop of the propagating coherent wavefront.

Moreover, the weak localization peak decayed with distance from the source; the total energy signals at all time instances collapsed along this curve except the propagating wavefront, which propagated along this long time limit decay curve.

**Figure 4:**
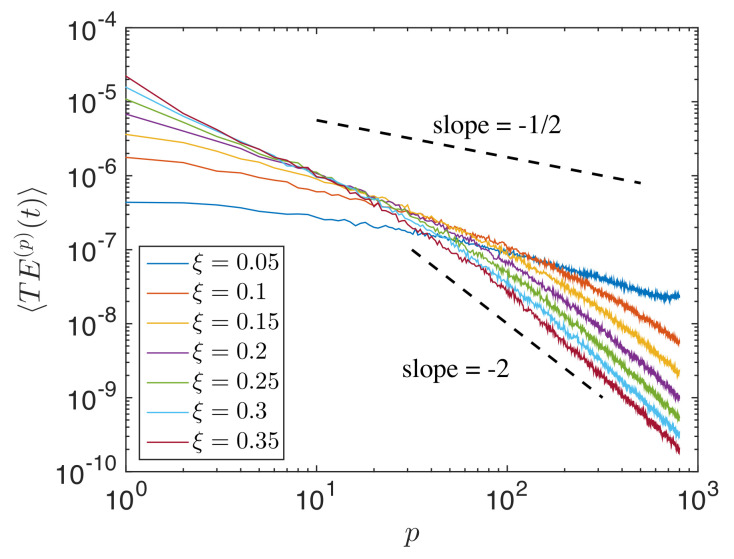
Power law relationship of the weak localization decay curve for different ξ, at later time t≅875.

**Figure 5:**
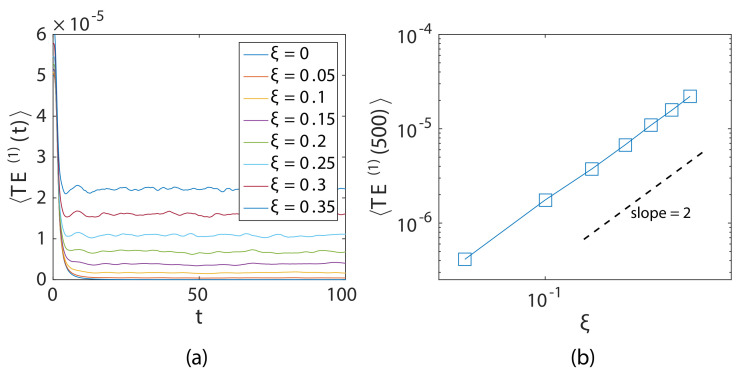
(**a**) Total energy (averaged over 500 ensembles) of the p=1 particle with time for chains with different ξ. (**b**) Residual energy localized in the first particle measured at t≅500.

Figure 4 is the log plot of the decay curve associated with weak localization for chains (ensembled total energy signal at t=875 per particle; measurements were taken by limiting the space interval (p≤800) to avoid propagating wavefront); a power law relationship can be observed from the figure. It was observed that the rate of decay increased with the increasing disorder parameter ξ of the chain, indicating a stronger weak localization decay curve with an increase in disorder. Figure 5a shows the total energy signal of particle p=1 (source) with time. The figure shows that after the initial impulse, the energy of the source particle decayed and became constant with very little fluctuations. This residual energy of the particle increased with increasing ξ. Figure 5b shows a power law relationship between the disorder ξ and the TE of the first particle at long time t=500.

#### 4.1.2. Diffusion

**Figure 6:**
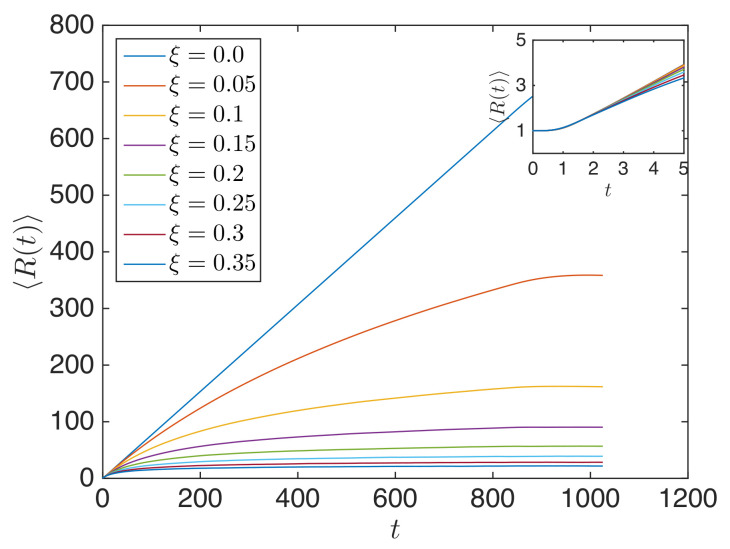
Position of the center of energy (〈R(t)〉) during impulse propagation for granular chains with different ξ.

The system used in the previous section (N=1024 particle long granular chain with $v_{o}=0.01$ imparted to the 1st particle) is used in this sub-section as well. Equation (37) was used to compute 〈R(t)〉 averaged over 500 ensembles. 〈R(t)〉 gave the averaged propagation of the center of energy, as plotted in Figure 6. It shows that initially, the center of energy did not propagate (as shown in the inset), during which the initial high-frequency impulse was self-demodulated [63] by the granular chain (in contrast to a Gaussian pulse [27]). After this short time interval, the center of energy propagated with the same speed for different disorder parameters. ξ=0.0 had linear (ballistic) propagation of the center of energy, whereas ξ>0 led to nonlinear propagation of the center of energy with propagation speed decreasing with increasing time [50]. Stronger disorder yielded a slower propagation speed with the increase in time. Unlike ξ=0.0, higher ξ resulted in the center of energy becoming confined in a finite space, and this confinement space was smaller for stronger disorder. This occurred because R(t) took into account both the weak localization occurring close to the source and the propagating wavefront.

**Figure 7:**
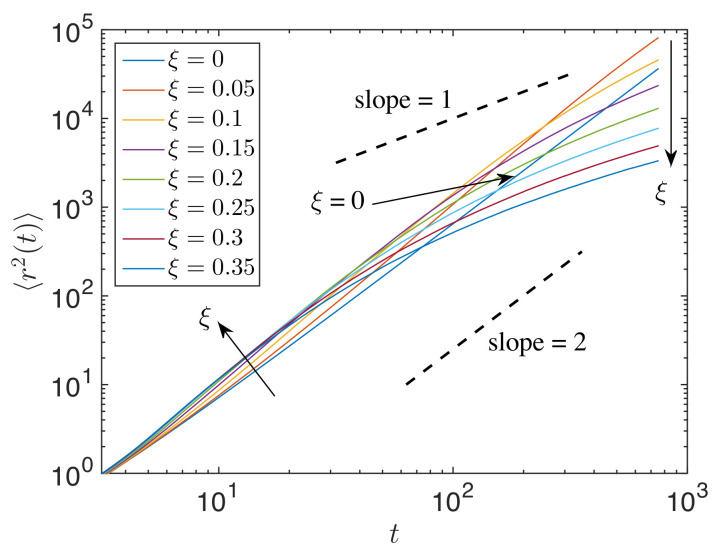
$\langle r^{2}\left(t\right)\rangle$ for different ξ in the log-log scale. The line is a guide to the eye. Arrows indicate the trend for ξ>0.

The mean squared width of the energy during impulse propagation $r^{2}\left(t\right)$ was computed using Equation (38), averaged over 500 realizations. Figure 7 displays $\langle r^{2}\left(t\right)\rangle$ for granular chains with different ξ. It is observed that for an impulse response, the energy propagation was slightly superballistic for low disorder parameters (e.g., ξ=0.05) and became nonlinear towards superdiffusive, diffusive, and then, subdiffusive for high disorder parameters.

#### 4.1.3. Center Particle Excitation

**Figure 8:**
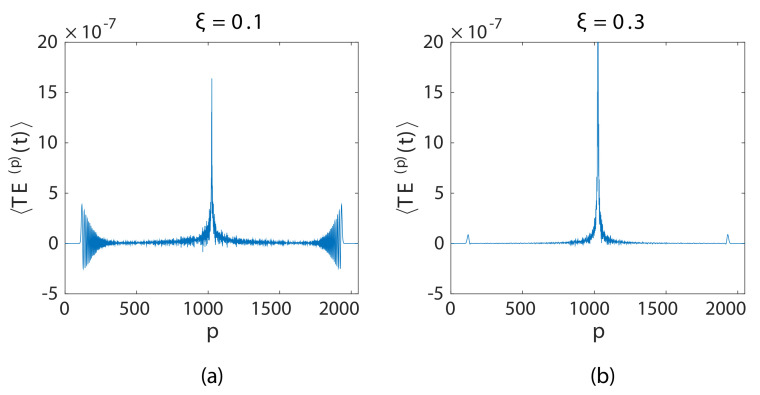
(**a**) The center pulse (p=1025) is imparted with $v_{o}=0.01$ initially towards the right. (**b**) The total energy signal is averaged over 500 ensembles at t≅750.

In order to ensure that the localization of energy during wave propagation is occurring near the source and is not a boundary effect, the effect of a different initial condition on impulse propagation in a granular chain is examined, p=1025th particle of N=2049 particle long granular chain was imparted with $v_{o}=0.01$. The time step for the computation Δt=0.2501, and $t_{max}=1024$. Figure 8 shows the total energy signal per particle at a particular instance of time t≅750 before the wave reached the end of the system for ξ=0.1 (Figure 8a) and ξ=0.3 (Figure 8b). It can be observed that the energy was localized around the source (the middle particle), and the two propagating wavefronts moved in the opposite direction. The figure is symmetric around the center particle, which confirms that we were in the linear regime, i.e., the tension and compression wave had the same speed.

### 4.2. Energy Propagation in Space and Time

**Figure 9:**
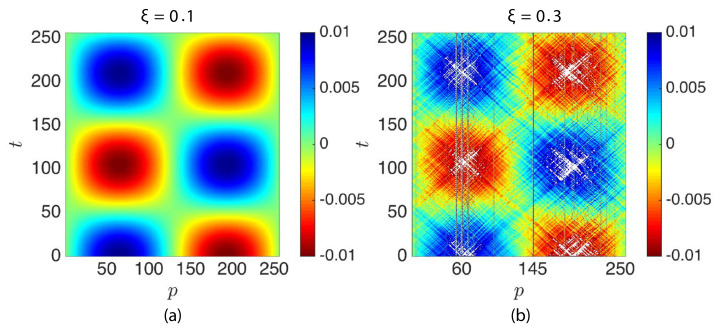
Sinusoidal standing wave (**a**) $u^{\left(p\right)}\left(t\right)$ (p=1 to 256) of an ordered granular chain (ξ=0.0). (**b**) $u^{\left(p\right)}\left(t\right)$ of a disordered granular chain (ξ=0.3), while color indicates the largest displacements; white patterns are amplitudes outside the color bar.

With the goal to understand the evolution of standing waves in time, an initial sinusoidal waveform ($u_{o}=u_{o}\sin\left(N\frac{2\pi p}{N+1}\right)$; Section 2.6) was imparted to an N+1=256 particle long granular chain with $u_{o}=0.01$ and different ξ. The evolution of displacement and energy responses of particles/elements were then analyzed. Figure 9 shows the evolving displacement of particles for the N=1 standing wave in an ordered (ξ=0.0) and a disordered chain (ξ=0.3). Figure 9a displays the particles (color signifies the displacement amplitude) performing a standing wave motion (ξ=0.0). However, in Figure 9b, it is observed that particles exhibited a perturbed standing wave motion of fluctuating low amplitude (color) in addition with traveling waves, clearly indicating that the disorder in the chains was disrupting the standing wave motion. It can also be observed that there were few localized high amplitude displacements shown by certain particles like p=60 and p=145; these particles were the lowest and the third lowest mass particles in the granular chain; in addition, p=60 was close to a peak of the standing wave.

**Figure 10:**
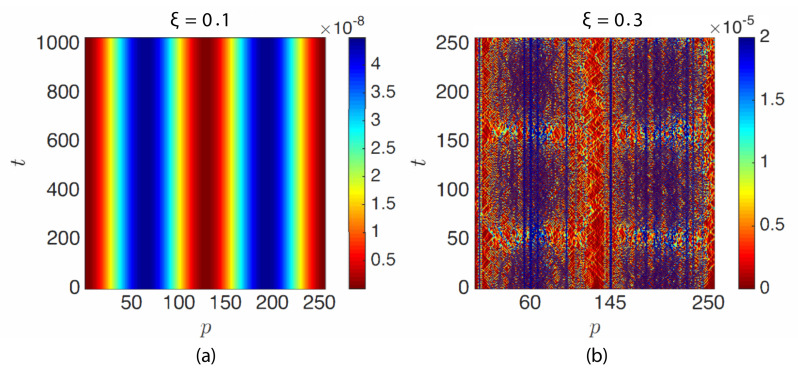
Total energy per particle $TE^{\left(p\right)}\left(t\right)$ (p=1 to 256) of (**a**) an ordered granular chain (ξ=0.0) and (**b**) a disordered granular chain (ξ=0.3), from the same data as in Figure 9.

Figure 10a shows the total energy of the particles for the ordered (ξ=0) and disordered (ξ=0.3) granular chains of Figure 9 (color represents the amplitude $TE^{\left(p\right)}\left(t\right)$). Unlike Figure 10a, Figure 10b exhibits localized high energy particles p=145 and p=60 (low mass particles), indicating the localization of energy due to the presence of disorder.

### 4.3. Energy Propagation Across Wave Numbers

**Figure 11:**
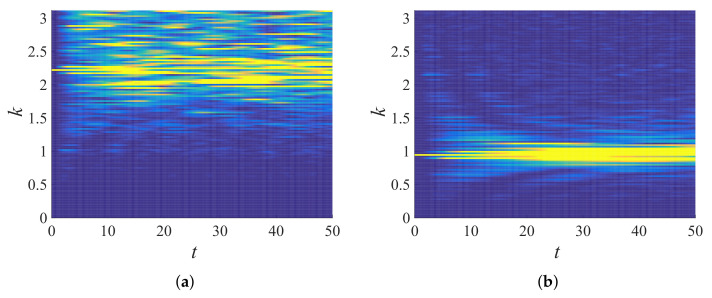
Total energy response ($TE^{\left(k\right)}\left(t\right)$) in wavenumber space plotted against time for a single realization of disordered granular chain (ξ=0.3) initially agitated with (**a**) $k_{ins}=2.159$ (N=90) and (**b**) $k_{ins}=0.981$ (N=38).

**Figure 12:**
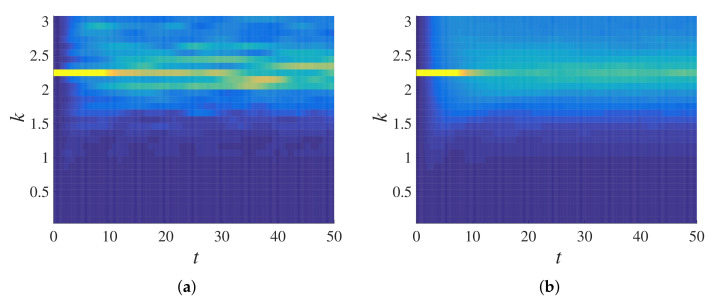
Binned total energy response of Figure 11a for (**a**) a single realization (${\hat{e}}^{\left(23\right)}\left(t\right)$) and (**b**) averaged over 100 ensembles ($\langle {\hat{e}}^{\left(23\right)}\left(t\right)\rangle$), with $k_{ins}=2.159$ (N=90), which gives bin number s=23.

Equation (43) was used to obtain total energy in the wavenumber space, then Equation (46) was used to bin the energy responses accordingly. The number of bins used for the computation here onwards is B=32 with a bandwidth Δk=π/32=0.0982. Figure 11 shows the temporal evolution of total energy in the wavenumber space for ξ=0.3 (the color scale in the plot is $TE^{\left(k\right)}\left(t\right)$), N=90 (Figure 11a) and 38 (Figure 11b). A peak was initially observed at the agitated wavenumber ($k_{ins}=N\frac{2\pi }{N+1}$); the peak decreased as the time progressed; the decay rate was lower for lower wavenumbers, which can be observed when Figure 11a and Figure 11b are compared. Figure 12 displays the binned total energy in the binned wavenumber space for $TE^{\left(k\right)}\left(t\right)$, from the same data as in Figure 11a. Figure 12b represents the same total energy response averaged over 100 ensembles, improving the quality of the signal (smoothness). Hence, in the following, ensemble averaged data are used, with averaging performed in *k*-space and binning performed thereafter.

**Figure 13:**
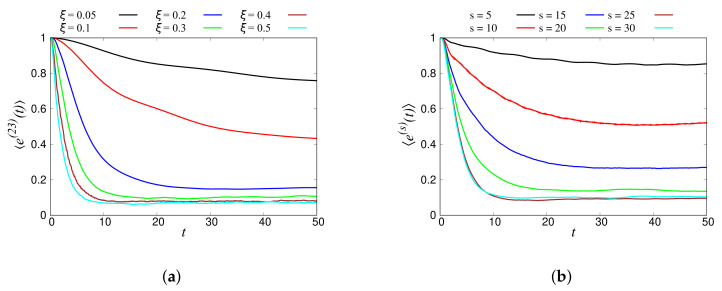
Normalized energy response of (**a**) the 23rd band for chains with different disorder, ξ, and (**b**) different input tones, N(=18,38,58,78,98,118), into bands, *s*, for the system with disorder ξ=0.3. The data shown here were averaged in *k*-space over 100 realizations.

In Figure 13a, the binned total energy response of the 23rd band is plotted for six different disordered chains (ξ = 0.05, 0.1, 0.2, 0.3, 0.4, and 0.5). The energy of the band decayed faster for higher disorder, and it reached its saturation faster than the systems with lower disorder. In fact, this observation was expected since higher disorder in the system led to faster loss of energy (attenuation) from the higher bands. Note that an ordered system ξ=0 did not show any decay of energy, since there was no “free” energy available for diffusion of energy across bands. Figure 13b shows the energy response of different agitated bands (s(N) = 5 (18), 10 (38), 15 (58), 20 (78), 25 (98), and 30 (118)) for a chain with disorder ξ=0.3. Higher bands *s* (higher wavenumber *k*) lost more energy than lower bands. That means that when a lower band was agitated, little energy was transferred to other bands, whereas the agitation of a higher band led to a significant energy transmission to other bands. The same behavior was seen for other disorders.

The number of particles of a simulation, i.e., the system size, can have a big influence on the simulation results. For this reason, two system sizes were chosen (*N* = 128, 512) along with the one used earlier (*N* = 256). Simulations with different sizes led to qualitatively and quantitatively similar results (data not shown).

### 4.4. Attenuation and Energy Transfer

Here, we first employ procedures explained earlier to obtain the components of the transfer matrix, $Q_{sr}$, before we interpret our observations. Finally, we validate the proposed master equation using the measured Q to solve the stochastic model that involves all bands, i.e., all wavenumbers (different from reduced order models that only consider lower eigenmodes).

**Figure 14:**
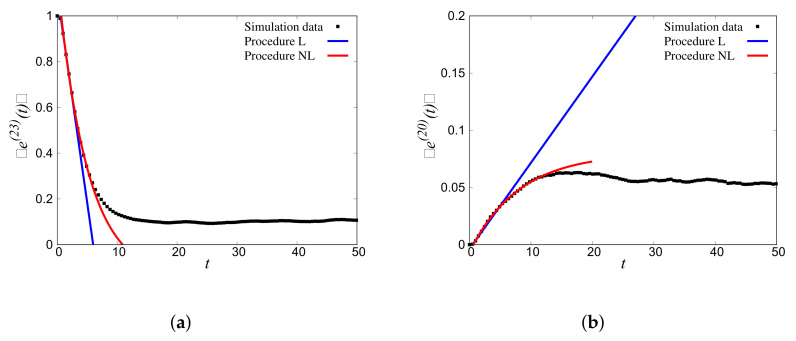
(**a**) Decaying binned total energy response of the initially agitated Bin 23 for $k_{ins}=2.159$ with the fits following the Linear (L) and Nonlinear (NL) procedures. (**b**) Increasing binned total energy response of Bin 20 receiving energy with the fits following the L and NL procedures. Only times from 2Δt to 8Δt were used for the linear fit and from 2Δt to 18Δt for the nonlinear fit, with Δt=1.

Figure 14a shows the ensembled decay rate of the bin, which contains the initially agitated wavenumber (bin s=23; $k_{ins}=2.159$) for disorder ξ=0.3; the decay was well captured by the fit (Section 3.4) through procedure L ($Q_{ss}^{\left(L\right)}=-0.18$) and procedure NL ($Q_{ss}^{\left(NL\right)}=-0.20$). The attenuation/damping coefficients obtained by linear and nonlinear fits were consistent.

In addition to the energy decay of bin s=23, Figure 14b displays the rise of energy in an adjacent bin (r=20), which received energy from bin s=23. The rise was well captured by the fitting procedures explained earlier (Section 3.5), where $Q_{sr}^{\left(L\right)}=0.0079$ and $Q_{sr}^{\left(NL\right)}=0.0081$.

The energy of the bin that was initially agitated decayed rather rapidly and achieved a steady state at $\langle {\hat{e}}^{\left(23\right)}\left(t\to \infty \right)\rangle \approx 0.10$. On the contrary, the energy of the bin, which received energy, started increasing much more slowly and achieved a different steady state at $\langle {\hat{e}}^{\left(20\right)}\left(t\to \infty \right)\rangle \approx 0.055$.

**Figure 15:**
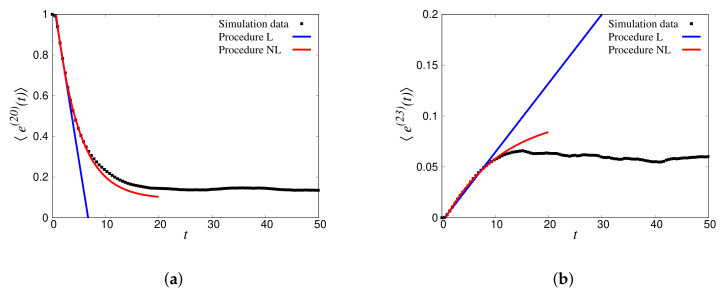
(**a**) Decaying binned total energy response of the initially agitated Bin 20 for $k_{ins}=1.86$ with the fits following the L and NL procedures. (**b**) Increasing binned total energy response of Bin 23 receiving energy with the fits following the L and NL procedures. Only times from 2Δt to 8Δt were used for the linear fit and from 2Δt to 18Δt for the nonlinear fit, with Δt=1.

A similar analysis is shown for the reverse case, i.e., source bin $s^{\prime }=20$, in Figure 15a, for $k_{ins}=1.86$, and the receiver bin $r^{\prime }=23$ in Figure 15b. The corresponding transfer matrix entries were: $Q_{s^{\prime }s^{\prime }}^{\left(L\right)}=-0.161$, $Q_{s^{\prime }s^{\prime }}^{\left(NL\right)}=-0.165$, $Q_{s^{\prime }r^{\prime }}^{\left(L\right)}=0.0071$, and $Q_{s^{\prime }r^{\prime }}^{\left(NL\right)}=0.0070$, with significantly different steady states at $\langle {\hat{e}}^{\left(20\right)}\left(t\to \infty \right)\rangle \approx 0.13$ and $\langle {\hat{e}}^{\left(23\right)}\left(t\to \infty \right)\rangle \approx 0.04$. This illustrates that the diagonal entries $Q_{ss}$ increased with *s*, while the non-diagonal entries, $Q_{sr}\approx Q_{s^{\prime }r^{\prime }}$, were approximately symmetric (within the error of the fits).

Note that in all cases, the fits were supposed to only catch the short time behavior, incrementally, not the steady-state limit.

Running simulations much longer ($t_{max}=500$ and 5000) confirmed that energy was conserved also on the long term, and that the system remained in its steady state achieved at the beginning of the simulation, t<50, i.e., no further transfer of energy between bands took place at very large times. However, note that systems with smaller disorder, as well as the lower bands, can take much longer to reach their steady states (this means that the fit ranges have to be adapted appropriately, decreasing with increasing disorder or band *s*, but also increasing with difference |s−r| (no further details shown here)).

**Figure 16:**
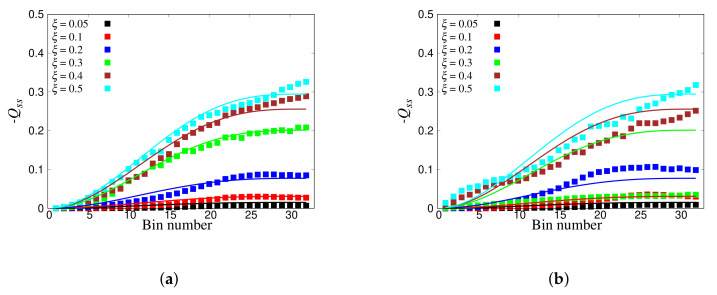
Diagonal components of Q obtained by the (**a**) L (Linear fit) procedure and (**b**) the NL (Nonlinear fit) procedure, for different disorders, ξ, as given in the inset. The symbols denote the direct fits, while the lines provide the normalization condition for $-Q_{ss}$.

The full transfer matrix Q can now be deduced from the wave-propagation simulations in disordered particle systems (in the ideal situation, without loss of generality, elastic and 1D). Using the set of $k_{ins}$ (where N=2,6,10,...,126, one mode from every bin, ΔN=4, Δk=π/32, hence encompassing all the 32 bins), using the fitting procedure explained in Section 3.4, the components of the transfer matrix were computed for ensembles of 100 disordered granular chains with ξ=0.05, 0.1, 0.2, 0.3, 0.4, and 0.5. In Figure 16a, symbols depict the diagonal elements of the Q matrix, directly computed by the L procedure. The diagonal elements $-Q_{ss}$ increased with increasing bin number (i.e., increasing $k_{ins}$) for different disorders. This can be attributed to the fact that higher bins/wavenumber energies decayed faster, as can be observed from Figure 13b. The loss of energy was also related to disorder, i.e., attenuation increased with increasing the sample disorder. In addition, we compare the $-Q_{ss}$ elements of Q directly derived from the nonlinear procedure (NL) in Figure 16b. Like for the linear function observations, $Q_{ss}$ increased with the increase of bin number and disorder.

**Figure 17:**
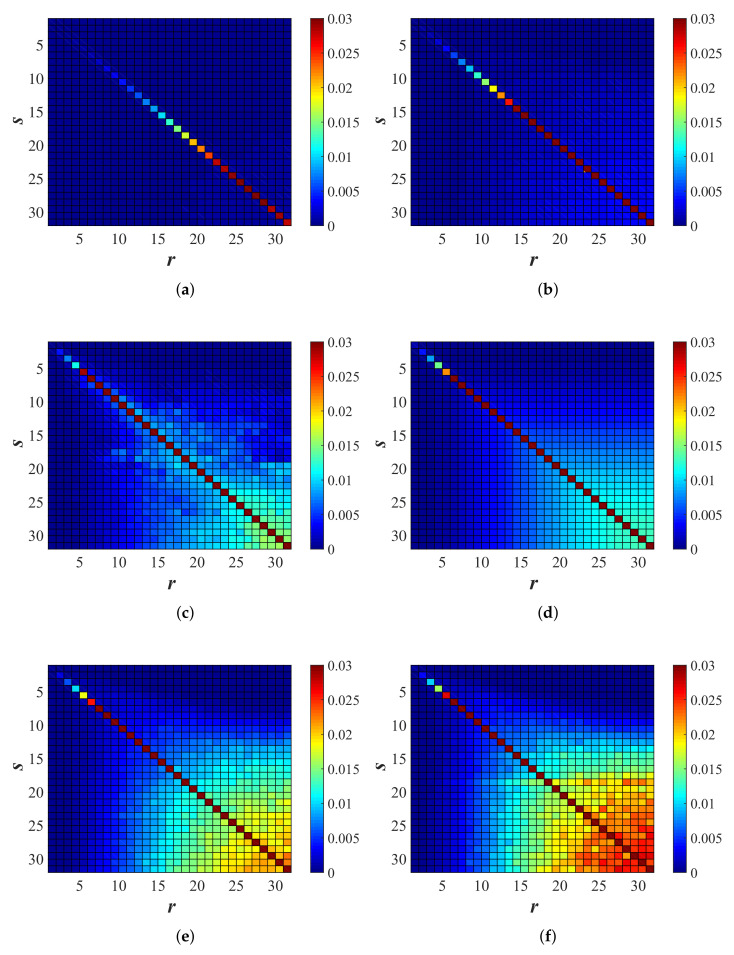
The transfer rate matrix $Q_{sr}$ computed through the linear procedure (Section 3.4) for different disorders: (**a**) ξ=0.1, (**b**) ξ=0.2, (**c**) ξ=0.3, and (**d**) the analytical expression (Equation (50)) for ξ=0.3, (**e**) ξ=0.4, and (**f**) ξ=0.5. The first index gave the source *s* (row) bin and the second the receiver *r* (column) bin. The non-diagonal elements $Q_{sr}$ were the increase rates of energy in bin *r* by receiving energy from bin *s*. The diagonal elements ($-Q_{ss}$) were the attenuation coefficients of the energy in band/bin *s* (no Einstein summation implied in ss).

Following the procedure explained in Section 3.4, also, the off-diagonal components of Q were computed. Figure 17 illustrates the color plots of Q computed by the linear procedure for six different disorder magnitudes. Based on previous observations [4,50], it was expected that the intensity of diagonal terms would keep increasing while the bin numbers increased, which means that higher bands would attenuate faster, i.e., transfer more energy to their neighbors in comparison to lower bins. On the non-diagonal, for low bands *r* and *s*, there were very small probabilities for transfer of energy to other bands, while for increasing *r* and *s*, the probability for energy-transfer to other bands increased; in particular, there was the most transfer of energy for the largest *r* and *s*. Note that the matrix obtained by fitting was not exactly symmetric.

The non-diagonal transfer matrix elements are approximately given by:

(50) $$Q_{sr}=q_{0}\left(\xi \right)\sin^{2}\left(\frac{\pi }{2B}\min\left(s,r\right)\right)\;,$$

which is perfectly symmetric, with maximal number of bins, *B*, and a prefactor $q_{0}$ increasing with disorder ξ, for example $q_{0}\left(\xi =0.3\right)\approx 0.013$.

The lines in Figure 16 provide the normalization condition: $-Q_{ss}=\sum_{s\neq r}Q_{sr}$, in reasonable agreement (±5−10%) with the direct measurements. The agreement/disagreement between different procedures, as well as the wiggles, showed the consistency, but also the imperfection of the automated fit procedure.

### 4.5. Stochastic Modeling—Energy Propagation in the Wavenumber

In the earlier section (Section 3.4), we developed the (reduced order) stochastic model based on the master equation (dropping the hat symbols for convenience):

(51) $$\frac{de^{\left(s\right)}\left(t\right)}{dt}=Q_{ss}e^{\left(s\right)}\left(t\right)+\sum_{s\neq r}Q_{rs}e^{\left(r\right)}\left(t\right)-d^{\left(s\right)}e^{\left(s\right)}\left(t\right)$$

that describes the evolution at time *t* of the energy $e^{\left(s\right)}\left(t\right)$ in the wavenumber band *s* at $k_{s}\pm \Delta k/2$, by transfer to all other bands, with rate $Q_{ss}$, or from all other bands *r* into *s* with rate $Q_{sr}$. Note that the sequence of indices in the transfer rates is relevant if $Q_{sr}$ is not perfectly symmetric. However, the non-symmetry observed was of the order of the uncertainty of the fits. The normalization condition $Q_{ss}=-\sum_{r\neq s}Q_{sr}$ guaranteed total energy conservation. The fitting procedures did not automatically achieve this, causing a small leakage of energy, but also showing that the procedures almost achieved the normalization. The last term in Equation (51) represents the frequency-dependent damping (energy dissipation, not attenuation), characterized by the damping rate $d^{\left(s\right)}$, which was zero in this research since the total energy in a chain was considered to be conserved.

Given any matrix Q, the evolution of energy with time can be easily modeled/integrated using the master equation in Equation (51). Thanks to the reduced order modeling in a master equation approach that combines many eigenmodes in the frequency bands, this solution is very efficient and much faster than any numerical solution of the full model.

**Figure 18:**
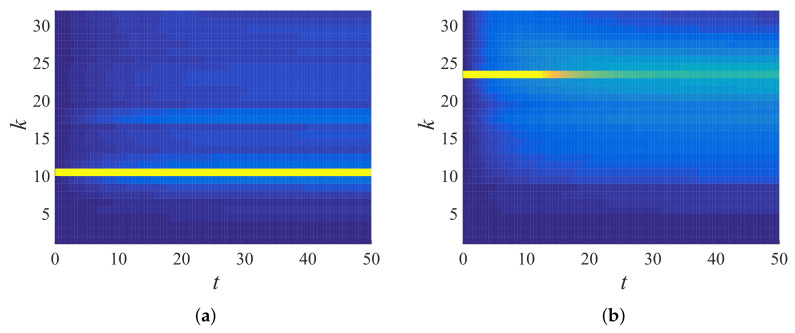
Frequency propagation of the (**a**) 10th and (**b**) 23rd bins computed using the Q matrix from Figure 17d, for ξ=0.3, in Equation (51) with $t_{max}=50$.

After measuring the components of the transfer matrix in the previous subsection, here, we tested the validity of the proposed master equation. Using Equation (51) and the Q matrix computed in Figure 17c (for the disordered system ξ=0.3), the frequency propagation of specific bins can be computed (the final purpose of the master equation formulation; here, this computation can serve as a cross-validation), which was done for two different bins, the 10th and 23rd, in Figure 18. Comparing the results using Equation (51) with earlier simulation results (Figure 11 and Figure 12), one can see that the model was in a perfect agreement with the simulations. As expected, the higher frequency bin (23rd bin) lost energy faster than the lower frequency bin (10th bin) to other frequency bins/bands; this indicates that the lower frequency passed and the higher frequency attenuated, a fundamental frequency propagation characteristic in disordered granular media, as observed earlier; see [4,50,64].

## 5. Conclusions

A mass-disordered granular chain with linearized Hertzian repulsive interaction forces between the granules is a model for disordered granular force chains (Section 2). Complementing our previous studies, here, we used for the first time the total energy evolution, not only displacement, velocity, or strain. Its simplicity allowed for analytical solutions and was used for studying the energy propagation characteristics using two types of initial conditions:

- Impulse initial conditions for studying energy propagation with distance (Section 2.5 and Section 4.1).
- Standing wave initial conditions for studying energy evolution in time by transfer across wavenumbers (Section 2.6, Section 4.2, Section 4.3, Section 4.4).

The latter case was used to calibrate the transfer matrix of a reduced stochastic model using the short time evolution of ensemble averaged energy in the wavenumber space with time. The master equation was then validated by the long time system evolution to its steady state, and an empirical analytical transfer matrix was proposed.

During impulse propagation, disordered systems, in Section 4.1, featured twin peaks in the total energy signal plotted against distance from the source; unlike ordered chains, which had only one peak. The peak near the source was attributed to localization due to disorder, whereas the second peak was the propagating coherent wavefront. The localization decay was observed to be invariant with time and exhibited a power law-like relationship with distance from the source, where the rate of decay increased with increasing disorder parameter (ξ). The wavefront decreased with distance from the source more strongly with increasing ξ. The center of average energy’s propagation speed decreased with ξ. Disorder led to a confinement of the fraction of the energy within a finite space. This resulted in a diffusive-like propagation of average energy. The ballistic propagation of energy in ordered chains appeared slightly superballistic for small disorder and became successively superdiffusive, diffusive, and subdiffusive with increasing disorder ξ. The localization effect and the diffusion model are interesting features of disordered media, which can be modeled and parametrized to understand and predict some of the microstructural material parameters from macroscopic wave propagation measurements.

Section 4.2 focused on the total energy evolution of standing waves with perfect sinusoidal initial conditions. In the real space, the energy becomes localized, around lower masses in the chain. In the wavenumber space, the energy in higher inserted wavenumbers decays faster/further than the energy in lower wavenumbers. Both trends increase with disorder. The master equation, introduced in Section 3.4, for the transfer of energies across wavenumbers (bands) modeled a disordered granular chain, easing the computational expense relative to the full model with all degrees of freedom, while maintaining the characteristics of energy transfer across all the wavenumbers, across the bands. Two procedures were compared, as a consistency check, to calibrate the components of the transfer matrix Q in the master equation, which quantified all transfer rates for a short time step. Applying Q again and again led to a long-time prediction of energy evolution in wavenumbers and time, in good agreement with the analytical data, but only after considering the additional mechanism of a basis energy per wavenumber band (which was not transferred).

The master equation acted as a stochastic model for modeling wave propagation in disordered media. Its core ingredient, the transfer matrix Q, can be improved with better statistics or better fitting curves. There are open issues in the fit procedure, like choosing the variable time intervals to be used for fitting the decay of the agitated bins or the rise in energy of the non-agitated bins. One option would be to estimate the appropriate time interval through the dispersion relation, according to both the initially inserted and the receiving wavenumber. Actually, an empirical analytical model as proposed based on the (noisy) calibrated transfer matrix.

The master equation, in its present form, did not contain nonlinear interactions between different wavenumber bands [64], and the frequency-dependent dissipation terms relevant for real materials were not studied (nor calibrated yet). As mentioned previously in Section 1, the master equation can now be used for continuum analyses associated with larger systems. It contains information about the microstructure in the form of components of the transfer matrix, but needs to be calibrated and validated experimentally.

Future work will focus on two- and three-dimensional packings of granular materials by employing the discrete element method for particle level numerical simulations. Furthermore, here, the investigation can be carried out not only on size/mass disorder, but also stiffness disorder, like previously in 1D, resembling functionality (material characteristic) where also different species, soft and stiff particles, can be mixed. To pursue the goal of predicting realistic system mechanics, as a first step, damping has to be added to the (reduced complexity) master equation so that the equation can be tested for different real materials; in experiments, we expect much stronger damping for softer (e.g., rubber) than for stiff (e.g. sand) particles. The master equation can then be calibrated separately for pure stiff (almost elastic) and pure soft (strong damping) samples. After defining the master equation for two (or more) types of materials, the challenge is calibrating also the transfer terms that quantify the transfer of energy between the species, where the characteristic time scales (rates) of the species can be highly different (fast for stiff vs. very slow for soft).

Another challenge for future research is to understand also nonlinear terms in the master equation, which can be added in the form of, e.g., mixed quadratic terms in energy, which have been shown to produce higher harmonics [64] and which might be needed in order to properly predict band-gaps, transmission bands, and possibly other nonlinear features due to interactions between different bands—in the presence of both single or multiple materials.

## Figures and Tables

**Figure A1:**
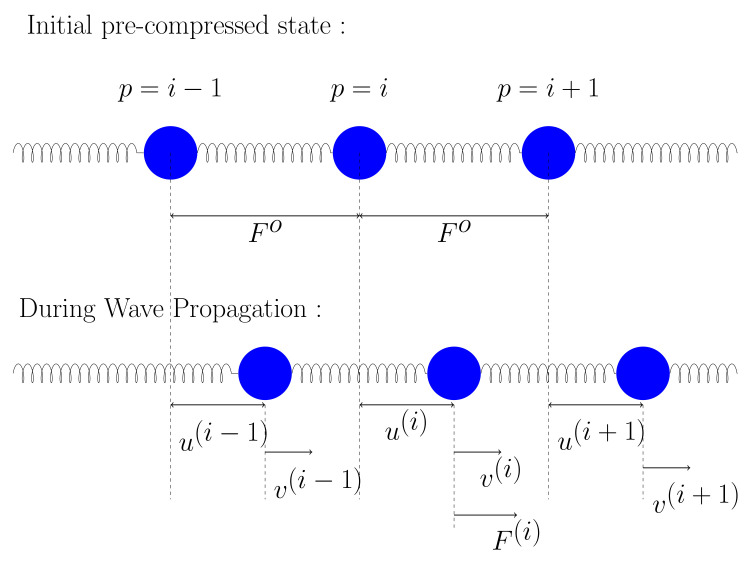
Particle chain for a simple triplet case to show the energy equations.

## Data Availability

Not applicable.

## Appendix A

Energy equations (kinetic, potential and total energy) are used for analyzing the properties of mechanical wave propagation across the granular chain. The matrix form of the equations assists in computing the signals both in real and wavenumber space with ease. Equations (30), (31), (33), and (34) are derived in this section using a three-particle system (i−1, *i*, and i+1). For better visualization, the pre-compressed granular chain is represented by a spring mass system, as shown in Figure A1. The springs are the interactions between the elements, resembling particle contacts.

### Appendix A.1. Kinetic Energy

The kinetic energy is zero at the initial pre-compressed static, equilibrium state. The kinetic energy of particle *p* during wave propagation is:

(A1) $$KE^{\left(p\right)}\left(t\right)=\frac{1}{2}b^{\left(p\right)}{\left(v^{\left(p\right)}\right)}^{2},$$

where $b^{\left(p\right)}$ and $v^{\left(p\right)}$ are non-dimmass and velocity, respectively. The kinetic energy can also be calculated by the use of matrices as in Equation (30),

(A2) $$\begin{array}{cc} \mathrm{KE} & =\frac{1}{2}M\left[v⊗v\right]=\frac{1}{2}Mvv^{T}\\ \; & =\frac{1}{2}\left(\begin{array}{ccccc} \ddots & \vdots & \vdots & \vdots & \vdots \\ \cdots & b^{\left(i-1\right)} & 0 & 0 & \cdots \\ \cdots & 0 & b^{\left(i\right)} & 0 & \cdots \\ \cdots & 0 & 0 & b^{\left(i+1\right)} & \cdots \\ \vdots & \vdots & \vdots & \vdots & \ddots \end{array}\right)\left(\begin{array}{c} \vdots \\ v^{\left(i-1\right)}\\ v^{\left(i\right)}\\ v^{\left(i+1\right)}\\ \vdots \end{array}\right)\left(\begin{array}{ccccc} \cdots & v^{\left(i-1\right)} & v^{\left(i\right)} & v^{\left(i+1\right)} & \cdots \end{array}\right)\\ \; & =\frac{1}{2}\left(\begin{array}{ccccc} \ddots & \vdots & \vdots & \vdots & \vdots \\ \cdots & b^{\left(i-1\right)}{\left(v^{\left(i-1\right)}\right)}^{2} & b^{\left(i-1\right)}v^{\left(i-1\right)}v^{\left(i\right)} & b^{\left(i-1\right)}v^{\left(i-1\right)}v^{\left(i+1\right)} & \cdots \\ \cdots & b^{\left(i\right)}v^{\left(i-1\right)}v^{\left(i\right)} & b^{\left(i\right)}{\left(v^{\left(i\right)}\right)}^{2} & b^{\left(i\right)}v^{\left(i\right)}v^{\left(i+1\right)} & \cdots \\ \cdots & b^{\left(i+1\right)}v^{\left(i-1\right)}v^{\left(i+1\right)} & b^{\left(i+1\right)}v^{\left(i\right)}v^{\left(i+1\right)} & b^{\left(i+1\right)}{\left(v^{\left(i+1\right)}\right)}^{2} & \cdots \\ \vdots & \vdots & \vdots & \vdots & \ddots \end{array}\right) \end{array}$$

The diagonal elements of this matrix give the kinetic energy of individual elements (Equation (A1)), and the total kinetic energy of the system is the trace of this matrix. The non-diagonal elements give the spatial velocity correlation of the elements with other elements in the system. For instance, $KE_{12}$ is the mass-weighed velocity correlation of the first element with the second element, but note that $KE_{12}\neq KE_{21}$.

### Appendix A.2. Potential Energy

During wave propagation, the relative potential energy is calculated as:

(A3) $$PE^{\left(p\right)}\left(t\right)=\left(PE_{\left(i-1,i\right)}+PE_{\left(i,i+1\right)}\right)/2$$

where $PE^{\left(p\right)}\left(t\right)$ is the potential energy of one individual particle, *p*, with $PE_{\left(i-1,i\right)}$ and $PE_{\left(i,i+1\right)}$ the potential energies due to the adjacent springs (contacts). The kinetic energy can also be calculated by the use of matrices as in Equation (30), The potential energy in matrix form is computed using Equation (33) as:

(A4) $$\begin{array}{cc} \; & \mathrm{PE}=-\frac{1}{2}K\left[u⊗u\right]=-\frac{1}{2}K{\mathrm{uu}}^{T},\\ \; & =-\frac{1}{2}\left(\begin{array}{ccccc} \ddots & \vdots & \vdots & \vdots & \vdots \\ \cdots & -\frac{3}{2}\left(\kappa_{\left(i-2,i-1\right)}^{2/3}+\kappa_{\left(i-1,i\right)}^{2/3}\right) & \frac{3}{2}\kappa_{\left(i-1,i\right)}^{2/3} & 0 & \cdots \\ \cdots & \frac{3}{2}\kappa_{\left(i-1,i\right)}^{2/3} & -\frac{3}{2}\left(\kappa_{\left(i-1,i\right)}^{2/3}+\kappa_{\left(i,i+1\right)}^{2/3}\right) & \frac{3}{2}\kappa_{\left(i,i+1\right)}^{2/3} & \cdots \\ \cdots & 0 & \frac{3}{2}\kappa_{\left(i,i+1\right)}^{2/3} & -\frac{3}{2}\left(\kappa_{\left(i,i+1\right)}^{2/3}+\kappa_{\left(i+1,i+2\right)}^{2/3}\right) & \cdots \\ \vdots & \vdots & \vdots & \vdots & \ddots \end{array}\right)\\ \; & \left(\begin{array}{c} \vdots \\ u^{\left(i-1\right)}\\ u^{\left(i\right)}\\ u^{\left(i+1\right)}\\ \vdots \end{array}\right)\left(\begin{array}{ccccc} \cdots & u^{\left(i-1\right)} & u^{\left(i\right)} & u^{\left(i+1\right)} & \cdots \end{array}\right) \end{array}$$

The diagonal elements of the matrix are:

(A5) $$PE_{PP}=-\frac{3}{4}\left[\kappa_{\left(i-1,1\right)}^{2/3}u^{\left(i-1\right)}u^{\left(i\right)}-\left(\kappa_{\left(i-1,i\right)}^{2/3}+\kappa_{\left(i,i+1\right)}^{2/3}\right)u^{\left(i\right)}u^{\left(i\right)}+\kappa_{\left(i,i+1\right)}^{2/3}u^{\left(i+1\right)}u^{\left(i\right)}\right].$$

Similar to the KE matrix, the non-diagonal elements of PE are non-symmetric and give the stiffness-weighted spatial displacement correlation of the elements with other elements in the system. In this study, however, only diagonal entries are used.
